# Supplementary material for: Influencing Factors and Symbiotic Mechanism of the Integration of Medical Care and Disease Prevention during the COVID-19 Pandemic: A Cross-Sectional Survey of Public Hospital Employees
Source: Int J Environ Res Public Health. 2022 Dec 23;20(1):241. doi: 10.3390/ijerph20010241 (PMC9819979; doi:10.3390/ijerph20010241)
Supplement: Supplementary file 1 [file ijerph-20-00241-s001.zip › ijerph-2091112-supplementary.pdf]

Survey Questionnaire of study on the mechanism of integration of medical and disease prevention in public hospitals

Dear Medical Staff.

Thank you very much for taking part in this questionnaire!

We are members of the project of Hangzhou Normal University, " Study on the mechanism of integration of medical and disease prevention in public hospitals". This questionnaire is an academic study and is conducted anonymously. The information you provide is for research purposes only and will not be used for any commercial purposes.

Please answer and tick the following questions according to your actual situation or feelings.

Thank you again for your support and cooperation.

1. Demographic characteristics

(1) Gender: ☐Male ☐Female

(2) Age: ________ Years

(3) Education: ☐ Associate college ☐ Undergraduate ☐ Master ☐ PhD

(4) Job title: ☐ Senior ☐ Primary

(5) Department: ☐ Clinical ☐ Public health ☐ Administration

(6) Occupation: ☐ Doctor ☐ Nurse ☐ Manager

(7) Working years:________ Years

2. Your evaluation on the effectiveness of the integration of medical and disease prevention in your public hospital

Items	Strongly agree	Agree	Neutral	Disagree	Strongly disagree
High level of awareness of the integration of medical and disease prevention	5	4	3	2	1
High level of acceptance of the integration of medical and disease prevention	5	4	3	2	1
High level of policy implementation of the integration of medical and disease prevention	5	4	3	2	1
High level of cross-departmental cooperation of the integration of medical and disease prevention	5	4	3	2	1

3. Your evaluation of the symbiosis unit in your public hospital

Items	Strongly agree	Agree	Neutral	Disagree	Strongly disagree
Good level of human resource allocation of the integration of medical and disease prevention	5	4	3	2	1
Good health personnel competency of the integration of medical and disease prevention	5	4	3	2	1
Rational multi-departmental set-up of the integration of medical and disease prevention	5	4	3	2	1
Rapid health emergency response capability of the integration of medical and disease prevention	5	4	3	2	1
Adequate financial resources of the integration of medical and disease prevention	5	4	3	2	1

4. Your evaluation of the symbiosis environment in your public hospital

Items	Strongly agree	Agree	Neutral	Disagree	Strongly disagree
A suitable policy environment of the integration of medical and disease prevention	5	4	3	2	1
A suitable communication environment of the integration of medical and disease prevention	5	4	3	2	1
A suitable technical environment of the integration of medical and disease prevention	5	4	3	2	1
A suitable data environment of the integration of medical and disease prevention	5	4	3	2	1

5. Your evaluation of the symbiosis model in your public hospital

Items	Strongly agree	Agree	Neutral	Disagree	Strongly disagree
Efficient human resource mechanism of the integration of medical and disease prevention	5	4	3	2	1
Efficient performance management mechanism of the integration of medical and disease prevention	5	4	3	2	1
Efficient information mechanism of the integration of medical and disease prevention	5	4	3	2	1
Efficient emergency mechanism of the integration of medical and disease prevention	5	4	3	2	1
Efficient training mechanism of the integration of medical and disease prevention	5	4	3	2	1
